# Test-adjusted estimation for pertussis incidence in greater Toronto, Canada, 1993–2006

**DOI:** 10.1186/s12879-025-12254-x

**Published:** 2025-12-12

**Authors:** Clara Eunyoung Lee, Amy A. Howe, Natalie J. Wilson, Alicia A. Grima, David N. Fisman

**Affiliations:** Dalla Lana School of Public Health, University of Toronto, 155 College Street, Toronto, M5T 3M7 Canada

**Keywords:** Epidemiology, Statistical data analysis, Bordetella pertussis, Vaccine-preventable diseases, Undiagnosed disease

## Abstract

**Background:**

Pertussis remains a major public health concern, particularly affecting young children. While most identified cases occur in this group, the burden among older children and adults who undergo less frequent testing is not well characterized.

**Methods:**

We analyzed pertussis testing and case data in the Greater Toronto Area from 1993 to 2006. We applied a meta-regression-based method for test adjustment by age and sex, estimating case counts in each demographic group as if they were tested at the same rate as the most tested group (< 1-year males).

**Results:**

Before adjustment, incidence was highest in the < 1-year group and declined with age, with the ≥ 80-year group having an incidence rate ratio (IRR) of 0.011 (95% CI: 0.006–0.020) relative to male children aged < 1 year. After adjustment, the 2-4-year group showed the highest relative incidence (IRR: 1.811, 95% CI: 1.117-2.938). The highest estimated underdiagnosed case rates were in the 2-4-year group at 13.69 per diagnosed case in males (95% CI: 5.913–21.467) and the 10-19-age group in females at 6.80 per diagnosed case (95% CI: 4.684–8.917).

**Conclusion:**

Our use of a novel test-adjustment method for estimating incidence suggests that while pertussis is most diagnosed in infants, it is substantially underdiagnosed in older age groups generally, but particularly so in preschool-aged children and the elderly. As undiagnosed infection in these populations may play a key role in sustaining transmission, this finding has implications for vaccine booster policy.

**Clinical trial number:**

Not applicable (not a clinical trial).

**Supplementary Information:**

The online version contains supplementary material available at 10.1186/s12879-025-12254-x.

## Background

Pertussis is a human respiratory infection caused by *Bordetella pertussis*. After attachment to the host respiratory epithelium, the bacteria release toxins that induce inflammation and damage the cilia of the upper respiratory tract [1]. Pertussis remains a significant threat to young children, as World Health Organization estimates that there are approximately 24.1 million cases of pertussis per year and 160,700 children under five years of age die of pertussis in a year [2]. Pertussis is highly contagious via respiratory aerosols, with up to 90% of household contacts and 50–80% of schoolroom contacts becoming infected after exposure [3]. It exhibits an overdispersed transmission pattern (i.e., a high fraction of transmission events can be traced to a small fraction of cases), which can result in superspreading events [4].

The implementation of the acellular vaccine in 1997–98, followed by the expansion of booster programs to adolescents in 1999 and to pregnant women in 2018, has proven highly effective [5]. Canada’s pertussis incidence declined to approximately 2.2 cases per 100,000 in 2010 after peaking of 31.8 per 100,000 in 1995 [6]. However, the resurgence observed in 2024, representing the highest activity since 2007, underscores the need for strengthened vaccination programs. The economic cost of pertussis is estimated at $79.6 to $241.3 million annually in Canada [6]. A machine learning study suggests significant underreporting, especially among adolescents and adults [7]. Using a modeling approach, McGirr et al. estimated that under-reporting increased with age, and approximately >95% of infections in children were caused by infections in persons with waning immunity following prior infection or vaccination [8].

Accurate assessment of pertussis burden is therefore critical for public health planning. While the infectious period can last up to three weeks, symptoms in adolescents and adults are often mild or absent, leading to lower testing rates and limited understanding of the true burden.

To address this, we developed a standardization-based meta-regression test-adjustment method [9], that adjusts for testing disparities across all demographic groups, generating incidence estimates that would be expected for subgroups if they were tested at the same rate as the most-tested subgroup. We previously applied this approach to COVID-19 epidemiology in Ontario, finding that while crude case incidence suggested an epidemic largely concentrated in older individuals, test-adjustment found that force of infection was likely driven largely by infection in younger adults (particularly younger men) and teens, whose infections were under-diagnosed due to limited testing [7]. We were able to validate this approach by comparing crude and test-adjusted cases to lagged COVID-19 deaths [10]. In this study, we applied this approach to pertussis epidemiology in the Greater Toronto Area of Ontario, Canada, to identify apparent changes in burden of infection by age and sex once after accounting for variability in testing.

## Methods

### Study material and definitions

The data used for this study has been the subject of previous work on pertussis epidemiology in the Greater Toronto Area (GTA), Canada from 1993 to 2006 [11]. GTA is the most populous metropolitan area in Ontario, located at the southern part of Ontario with a population of 5,113,149 in 2006, distributed over an area of 7,125 km^2^. Pertussis is subject to mandatory reporting to regional public health agencies when a case was confirmed with either *B. pertussis* isolation or a positive polymerase chain reaction (PCR) assay regardless of symptomatology. Data included here cover pertussis culture and PCR tests of respiratory specimens, including sputum, throat swabs, and nasal discharge.

PCR for pertussis was introduced in January 1999. Initial specimens were double tested by both culture and PCR. When individuals were tested using both methods, this was considered a single test. As reported elsewhere [11] during the period under study all pertussis cases in the GTA were diagnosed at one of two laboratories: the Ontario Public Health Laboratory or the laboratory of the Hospital for Sick Children. As such, this database represents a complete record of pertussis cases and testing during the period under study.

Age-specific population data were extracted from the Statistics Canada Census for the years 1993 to 2006 [12]. The study population was stratified into nine groups: <1 year (infants), 1 year (toddlers), 2–4 years (preschool-aged children), 5–9 years (children), 10–19 years (adolescents), 20–39 years (young adults), 40–59 years (middle-aged adults), 60–79 years (seniors), and ≥ 80 years (elderly). These age groups were selected to reflect immunization schedules and epidemiologically relevant age bands. For the < 1 year, 1 year, and 2–4-year groups, estimates were derived from the 0–5 years age band using population assumptions based on typical age structure.

Population estimates for individuals aged 90 years and older were unavailable for the years 1993–1999. To address this, age-specific population counts for the 90–94, 95–99, and ≥ 100-year age subgroups were retrospectively estimated using observed annual growth rates from 2000 to 2006, stratified by sex. These estimates were then collapsed into a single ≥ 80-year group for analysis. Records with implausible ages (> 110 years or < 0 years) were excluded due to concerns about data validity. Cases without recorded biological sex were also excluded.

### Statistical analysis

Negative binomial regression models were constructed using age- and sex-specific annual total case and test counts, along with corresponding population denominators. Reported daily data were aggregated into annual totals to minimize instability associated with zero counts, particularly in older age groups. The < 1-year group served as the reference category for comparing the incidence rate distributions. Negative binomial regression models were constructed using age- and sex-specific annual total case and test counts, and relevant population denominators. To quantify disparities in disease detection and testing, we calculated standardized infection ratios (SIRs) and standardized testing ratios (STRs). These were defined as the observed case or test rates in each age-sex group divided by the rate in the reference group (males aged < 1 year old). To address zero values in model offsets per-test models, a constant of 0.000005 was added to all cells.

Confidence intervals for the ratios were derived using the standard error of the natural logarithm of the ratio (ln[ratio]), calculated as follows:

$$\begin{aligned}\:\mathrm{S}\mathrm{E}\left[\mathrm{l}\mathrm{n}\right({\mathrm{S}\mathrm{I}\mathrm{R}}_{\mathrm{i}\mathrm{j}}\:\mathrm{o}\mathrm{r}\:{\mathrm{S}\mathrm{T}\mathrm{R}}_{\mathrm{i}\mathrm{j}}\left)\right]\:=&\:\surd\:\left[\right(1/{\mathrm{a}}_{\mathrm{i}\mathrm{j}})\:+\:(1/{\mathrm{b}}_{\mathrm{i}\mathrm{j}})\:\\&+\:(1/{\mathrm{c}}_{\mathrm{i}\mathrm{j}})\:+\:(1/{\mathrm{d}}_{\mathrm{i}\mathrm{j}}\left)\right]\end{aligned}$$

where *a* is the number of cases or tests in the subgroup; *b* is subgroup population; *c* is total number of cases or tests in the reference group; *d* is reference group population; and *i* and *j* represent age, sex, respectively.

We then used meta-regression models to examine the influence of age and sex on SIRs and STRs. This model followed the form:

ln(SIR_ij_) = α + β_ij_x_ij_, where α represents the model intercept, and β terms represent coefficients for the *i*th age and *j*th sex.

Test-adjusted SIRs were used to back-calculate and derive the hypothetical incidence that would be observed if all demographic groups were tested as frequently as the reference group. These test-adjusted incidences were used to approximate the potential burden of underdiagnosed pertussis in groups with lower testing rates. Using the test-adjusted case counts, we refitted the negative binomial models to assess whether the relationship between age, sex and incidence changed after accounting for differences in testing intensity. An additional post-adjustment model was fitted, including sex, age group, and their interaction term as covariates, to explore effect modification by sex.

Data cleaning was performed using R (version 4.4.2), and all statistical analyses were conducted in STATA (version 18.5; StataCorp, College Station, TX, USA). The study was approved by the Research Ethics Board of the University of Toronto (protocol number 48684). Informed consent was waived because the study used pre-collected anonymized datasets, and neither patients nor the public were involved in the conduct of this research. This study was carried out in accordance with institutional guidelines and the principles of the Declaration of Helsinki. No tissue samples were analyzed and no experiments were performed.

## Results

### Crude and relative incidence

**Fig. 1:**
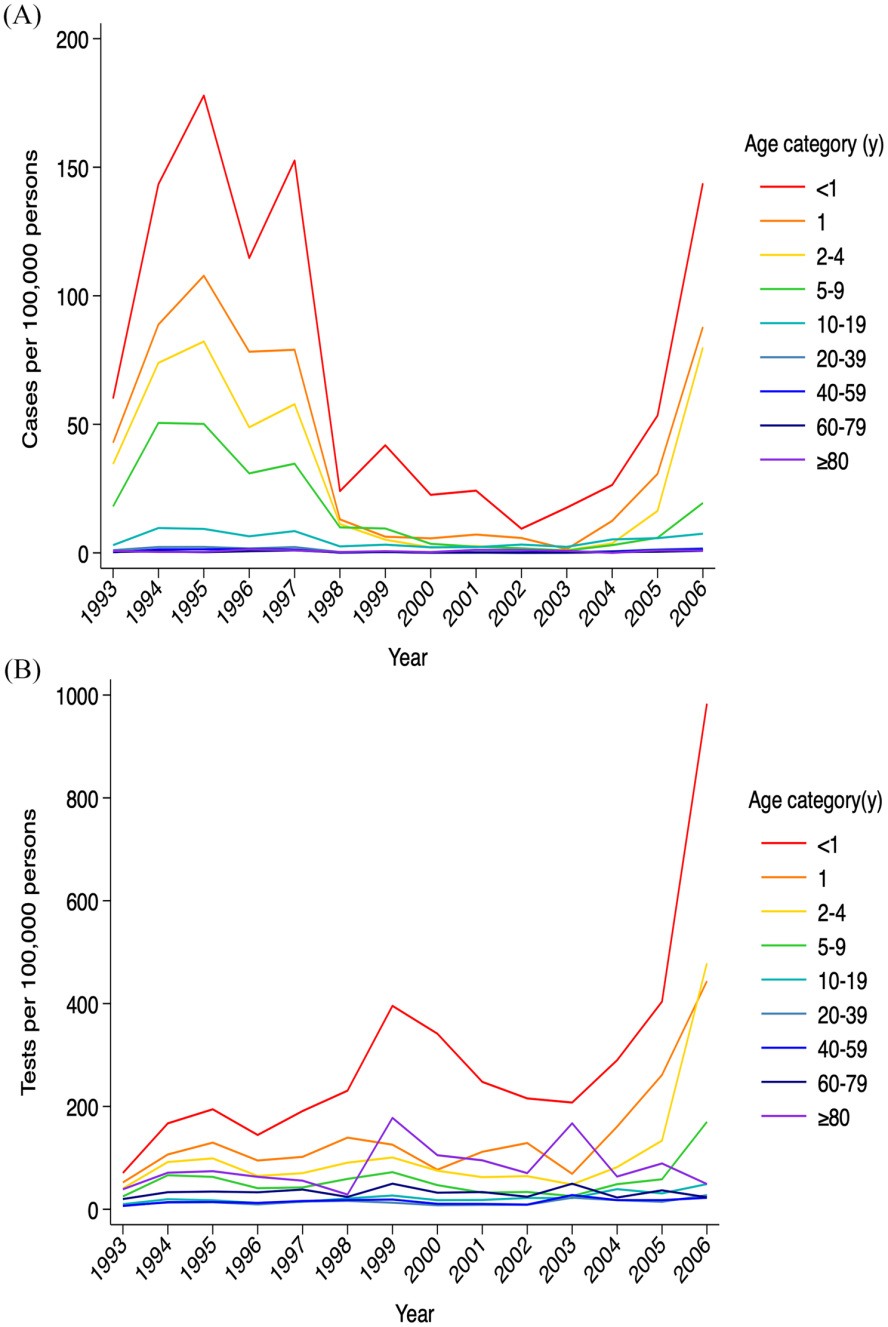
Trends in pertussis cases and testing in Greater Toronto Area, Ontario, Canada, between 1993 and 2006. Annual pertussis cases and tests by sex and age group were divided by the respective population and expressed per 100,000 persons. A rainbow-like color gradient (red to purple) was applied to represent age groups from < 1 year to ≥ 80 years, with each hue corresponding sequentially to increasing age. The annual incidence shows an overall decreasing trend with advancing age, with the < 1-year group consistently remaining the highest each year (**A**). The annual test rate increased steadily, with a sharp rise in 2006. It was also highest in the < 1-year group, although the ≥ 80-year group showed a substantial rate (**B**)

Out of 51,135 collected pertussis tests, 8,023 cases were identified. The highest overall crude incidence occurred in 1995 (13.14 cases per 100,000 persons), followed by 12.25 cases per 100,000 persons in 1994. While case counts decreased during the 2000s, there was a resurgence in 2006, reaching 8.32 cases per 100,000 (Fig. 1).

**Fig. 2:**
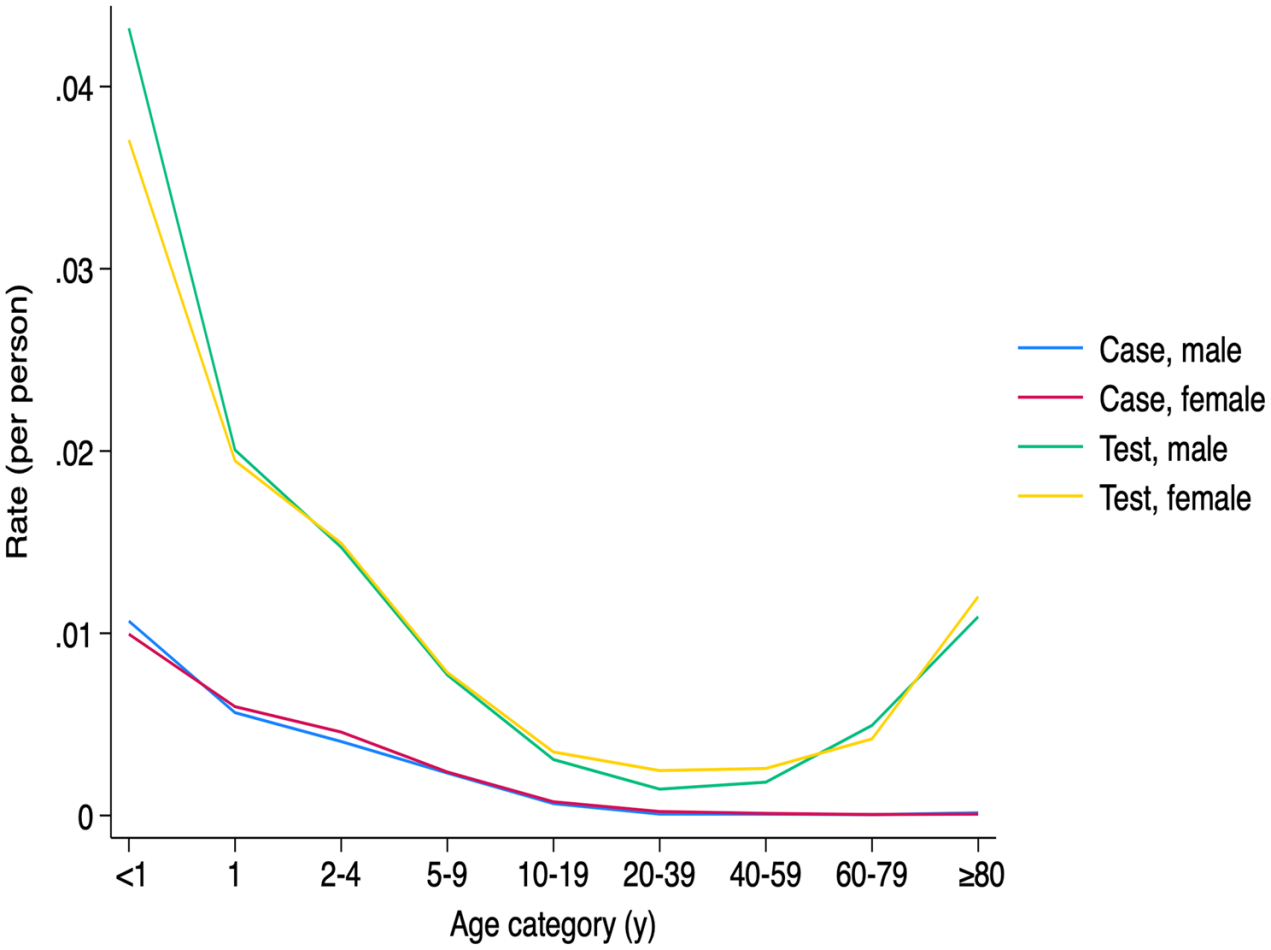
Overall pertussis case rates and test rates by sex and age category. This figure displays combined case and test rates by sex and age group. The total number of cases and tests was aggregated by sex and age category, then divided by the average population of each respective age group. The overall case rate for males (blue), and females (red) declines with age. The overall test rate for male (green) and female (yellow) follows a U-shaped pattern, with a trough in the 20-39-year age group and an increase in the older age groups

The < 1-year age group consistently exhibited the highest crude incidence (average 76.22 cases per 100,000 in males and average 71.10 per 100,000 in females). Incidence declined steadily with age, reaching 0.35 in males and 0.39 in females aged 60–79. It increased in those aged ≥ 80 years, to 1.05 in males and 0.55 in females (Fig. 2).

Relative differences by age are presented in Table 1 via incidence rate ratios (IRRs) from a negative binomial regression. Compared to the < 1-year group, IRRs declined with age, from 0.550 (95% confidence interval [95% CI]: 0.338–0.895) in the 1-year group, reaching 0.005 (95% CI: 0.003–0.009) in the 60–79 group, with a slight increase to 0.011 (95% CI: 0.006–0.020) in those ≥ 80 (*p* = 0.016 for the 1-year group, *p* < 0.001 for all other groups). No significant difference was found between sexes (IRR: 1.175, 95% CI: 0.921–1.498).

**Table 1:** Negative binomial models of pertussis incidence, testing incidence and test-adjusted incidence

Covariates	Crude incidence per population		Testing rate per population		Test-adjusted incidence per population	
	Incidence rate ratio (95% CI)	*P* value	Test rate ratio (95% CI)	*P* value	Incidence rate ratio (95% CI)	*P* value
Female sex	1.175 (0.921–1.498)	0.195	1.068 (0.961–1.188)	0.223	1.680 (1.305–2.164)	< 0.001
Age group						
< 1 y	1	-	1	-	1	-
1 y	0.550 (0.338–0.895)	0.016	0.499 (0.399–0.626)	< 0.001	0.805 (0.496–1.308)	0.381
2–4 y	0.408 (0.252–0.661)	< 0.001	0.371 (0.297–0.464)	< 0.001	1.811 (1.117–2.938)	0.016
5–9 y	0.217 (0.134–0.353)	< 0.001	0.206 (0.165–0.258)	< 0.001	0.907 (0.560–1.469)	0.690
10–19 y	0.073 (0.045–0.118)	< 0.001	0.081 (0.065–0.101)	< 0.001	0.337 (0.207–0.546)	< 0.001
20–39 y	0.014 (0.009–0.023)	< 0.001	0.051 (0.041–0.064)	< 0.001	0.028 (0.017–0.046)	< 0.001
40–59 y	0.010 (0.006–0.016)	< 0.001	0.057 (0.045–0.071)	< 0.001	0.012 (0.007–0.020)	< 0.001
60–79 y	0.005 (0.003–0.009)	< 0.001	0.126 (0.101–0.158)	< 0.001	0.005 (0.003–0.008)	< 0.001
≥ 80 y	0.011 (0.006–0.020)	< 0.001	0.301 (0.240–0.378)	< 0.001	0.051 (0.030–0.084)	< 0.001
Year	0.944 (0.920–0.968)	< 0.001	1.067 (1.054–1.081)	< 0.001	0.936 (0.913–0.959)	< 0.001

Abbreviation: 95% CI, 95% confidence interval

### Descriptive epidemiology of testing

Testing frequency increased over time, rising from 14.25 tests per 100,000 in 1993 to 36.81 in 2005, with a sharp increase to 66.85 in 2006. The highest testing rates were in the < 1-year group (referent), decreased up to ages 20–39, followed by a increase in older age groups (*p* < 0.001 for all groups, Table 1). There was no significant sex difference in testing (Test Rate Ratio: 1.068, 95% CI: 0.961–1.188). Testing increased over time year-over-year (Test Rate Ratio: 1.067, 95% CI: 1.054–1.081, *p* < 0.001).

### Negative binomial model after test-adjustment

**Fig. 3:**
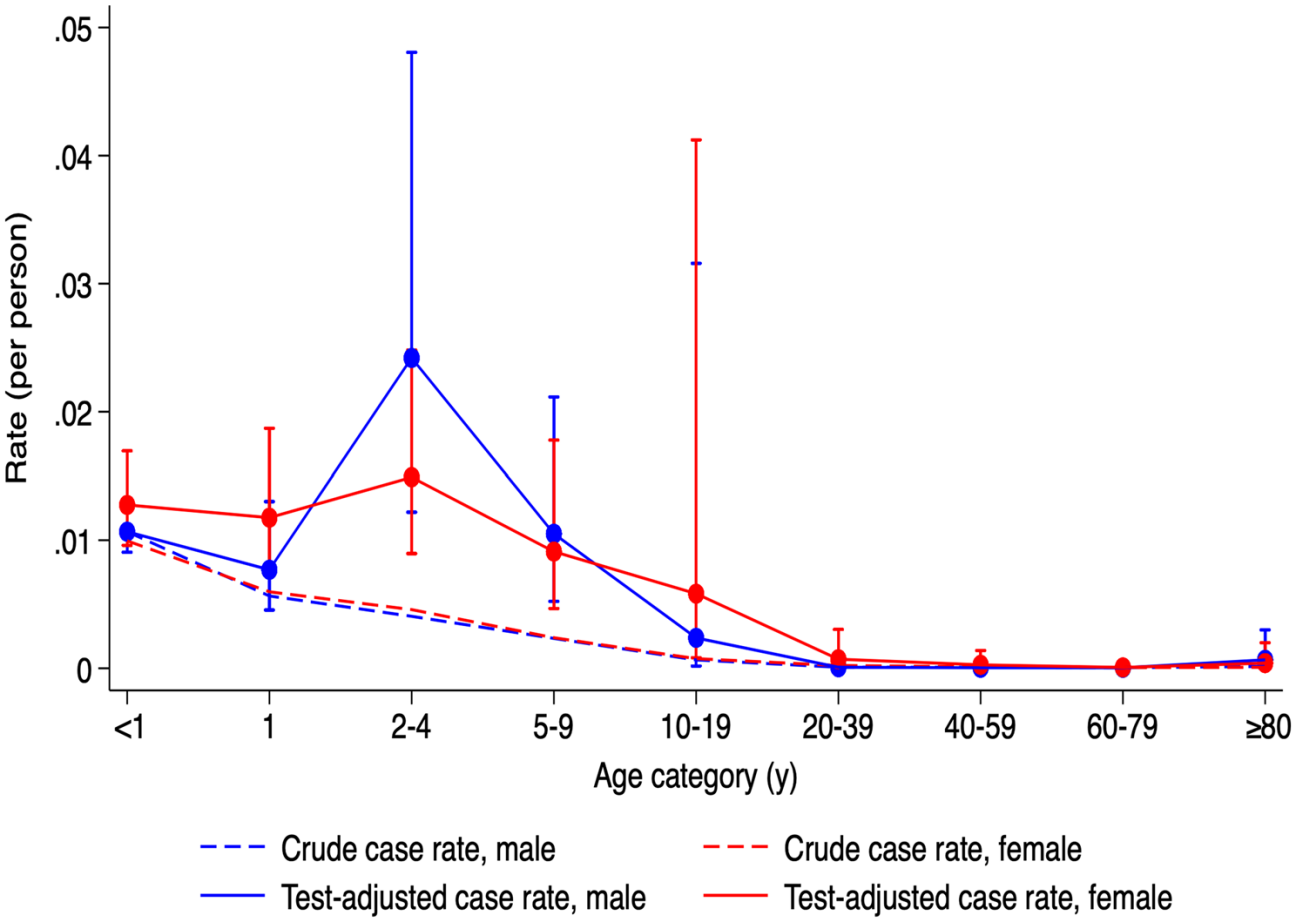
Crude and test-adjusted estimates of pertussis incidence in Ontario, Canada between 1993 and 2006. The lower, dashed lines show observed overall case rates by age and sex (blue for males, red for females), calculated as total number of cases divided by the average population size. The upper, solid lines show test-adjusted incidence estimates using standardized infection ratios, referencing the < 1-year male group (the most frequently tested). Bands indicate 95% confidence intervals

To adjust for variations in testing intensity, a negative binomial regression model was constructed with test volume as a covariate. In this model, the 2-4-year group had a significantly elevated IRR of 1.811 relative to < 1-year-olds (95% CI: 1.117–2.938, *p* = 0.016). Other age groups also showed elevated IRRs compared to the unadjusted model (Table 1; Fig. 3). The 1-year and 5-9-year groups were not significantly different from the reference group IRRs: 0.805 and 0.907, respectively). The lowest adjusted case rate was seen in the 60–79 and 40–59 age groups.

### Meta-regression derived test-adjusted incidence

Meta-regression using log-transformed STRs and SIRs demonstrated a significant increase of 1.300 in SIR per 1-unit increase in STR (95% CI: 1.172–1.426, *p* < 0.001). Subsequently, separate meta-regression models were conducted for each age category, stratified by sex.

**Fig. 4:**
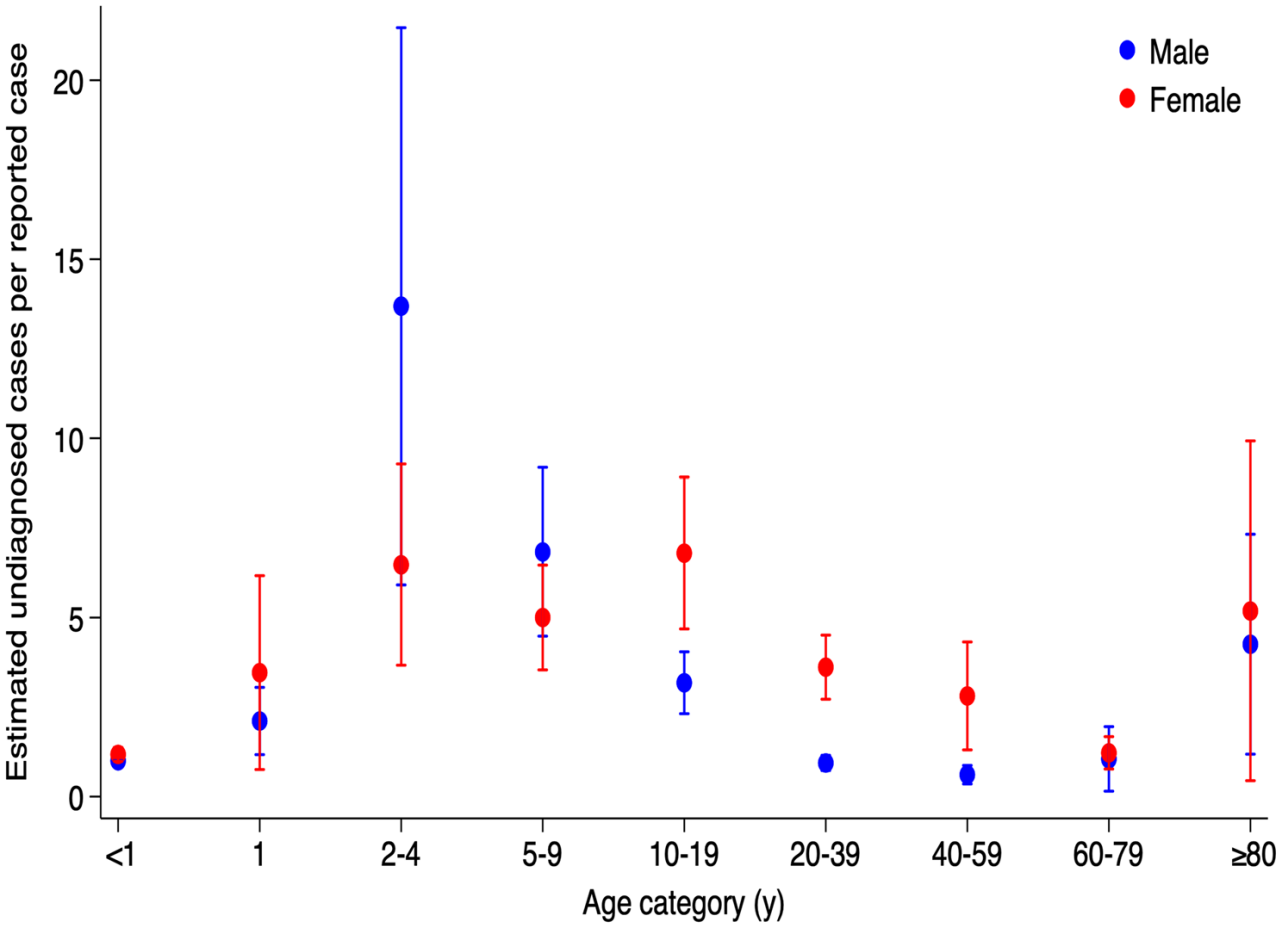
Estimated number of undiagnosed pertussis cases per reported case, by sex and age, in the Greater Toronto Area, Canada between 1993 and 2006. This figure represents the estimated ratio of undiagnosed to reported cases, calculating by adjusting reported cases for testing rates across sex and age groups. Each point indicates the estimated number of undiagnosed cases per each reported case, with 95% confidence intervals shown as vertical bars. Blue and red dots represent estimates for males and females, respectively

Estimated underdiagnosis was highest in 2-4-year-olds, at 13.69 undiagnosed cases per diagnosed male case (95% CI: 5.913–21.467) and 6.48 in females (95% CI: 3.670–9.281). This was followed by the 5-9-year-olds (6.84 in males [95% CI: 4.482–9.188]; 5.00 in females [95% CI: 3.539–6.466]) (Fig. 4, Supple. Table 1). In the ≥ 80-year group, underdiagnosis estimates were 4.26 for males (95% CI: 1.185–7.329) and 5.18 for females (95% CI: 0.444–9.924). In females, the 10-19-year-olds also showed high underdiagnosis (6.80, 95% CI: 4.684–8.917), as did females in the 20–39 and 40–59 groups compared to males.

After test-adjustment, a significant female predominance emerged in pertussis incidence. This was most pronounced in the 20–39 group (IRR: 7.295, 95% CI: 2.872–18.533, *p* < 0.001) and 40–59 group (IRR: 5.503, 95% CI: 2.121–14.281, *p* < 0.001) when age-by-sex interaction terms were included (Table 2).

**Table 2:** Negative binomial regression of test-adjusted incidence of pertussis per population to examine interaction between sex and age category

Covariates	Incidence rate ratio	95% conf. interval	*P* value
Female*<1y	1.199	0.625–2.301	0.584
Female*1 y	1.279	0.508–3.216	0.601
Female*2–4 y	0.515	0.206–1.290	0.157
Female*5–9 y	0.725	0.290–1.813	0.491
Female*10–19 y	2.046	0.818–5.125	0.126
Female*20–39 y	7.295	2.872–18.533	< 0.001
Female*40–59 y	5.503	2.121–14.281	< 0.001
Female*60–79 y	1.443	0.522–3.94]91	0.479
Female*≥80 y	0.510	0.194–1.341	0.172

Note: The model included sex, age group, and the interaction between sex and age group as covariates

## Discussion

This study estimated pertussis incidence using a test-adjustment method that revealed a substantial burden of underdiagnosis. Before adjustment, the IRR in the < 1-year group was 89 times higher than in the ≥ 80-year group. After adjusting for test intensity, this difference decreased to 20-fold. Notably, the 2-4-year group’s IRR increased from 0.408 to 1.817, indicating a significantly higher incidence under-testing was accounted for. This group also had the highest estimated burden of undiagnosed pertussis—13.7 undiagnosed cases per reported case in males and 6.5 in females. The 5-9-year age group had the second-highest IRR (0.905), statistically comparable to the < 1-year group.

Crude data aligned with previous Canadian reports, where infants showed the highest incidence, particularly during the outbreaks between 1993 and 1998, followed by declining incidence until 2005 with persistent < 1 year predominance [13]. Another report (2005–2019) showed that children aged 1–4 years remained a significant share of cases after infants [14]. The U.S. CDC surveillance also reported rising pertussis rates beyond infancy, with rates increasing from 47.89 to 59.00 per 100,000 in infants and 5.57 to 16.31 in 1-6-year-olds between 1990 and 2019 [15]. Whether this reflects growing clinical awareness or broader diagnostics remains unclear. It is concerning that this trend continued upwards globally including developed countries, until the COVID-19 pandemic disrupted disease patterns [16, 17].

The elevated undiagnosed burden in 2-4-year-olds highlights the importance of vaccine uptake. The age categories used in this study were designed based on the diphtheria, tetanus, and acellular pertussis-containing vaccines (DTaP) vaccine schedule, which includes four doses at 2, 4, 6, and 18 months of age, followed by a booster between 4 and 6 years. Ideally, children aged 2–4 years should be protected if vaccination coverage were adequate. However, despite the availability of a safer and effective acellular vaccine, the 2002 National Immunization Coverage Survey reported only 75.2% coverage for the full four-dose series in Canada [18]. By 2023, coverage had dropped further to 72.1% according to the STARVAX surveillance [19]. These suggest room for improvement in vaccine acceptance, particularly as children in this age group not only bear a significant disease burden but may also serve as an infection source to their younger siblings. Their extensive contact with caregivers and older children likely amplifies their role in transmission.

Elderly individuals also exhibited substantial underdiagnosis, with an estimated 4.3 undiagnosed male cases and 5.1 female cases per reported case. The contribution of epidemics among the elderly has also been reported in other studies [20]. While previous studies focused on individuals aged ≥ 60 years, our findings point to a particularly high burden in those 80 and older. Many elderly individuals reside in long-term care facilities, where diagnostic delays may lead to outbreaks. Given that only 33% of Canadian adults have received a pertussis booster in adulthood, the risk in older populations remains substantial [14].

After test adjustment, females had significantly higher pertussis incidence than males (IRR: 1.674). The age-sex interaction model revealed that this was most pronounced in the 20–39 and 40-59-year age groups. This may reflect greater exposure due to caregiving roles. The higher number of undiagnosed cases in these groups suggests that more thorough contact tracing could be beneficial.

This study offers several strengths. By adjusting for the number of individuals tested, we allow more accurate comparisons of incidence across demographic groups. Test-adjusted incidence captures potential differences in disease severity, healthcare access, and test-seeking behavior not reflected in crude testing rates. Targeted testing of high-risk groups can artificially inflate positivity rates, while broad testing can dilute them. This makes raw positivity rates problematic for comparisons across time or between populations with differing testing strategies. Simply put: more test, more case. These limitations were key drivers for developing our adjustment approach.

For a policy perspective, this method provides a practical and cost-effective tool for jurisdictions where detailed epidemiological studies are not feasible due to limited capacity, infrastructure, or political considerations. Similarly, sero-epidemiological studies are often hampered by antibody waning, imperfect sampling, the complexity of distinguishing vaccine-derived antibody from infection-derived antibody, and delays between infection and seroconversion. In contrast, our method offers a scalable and pragmatic alternative that leverages existing case and testing data to adjust for differential testing rates. It can be readily applied in various settings to improve the accuracy of case estimates and support more informed public health decision-making—especially in jurisdictions where more resource-intensive surveillance methods are not feasible. Our meta-regression model is designed specifically to adjust for differences in testing intensity across subgroups. This approach allows us to calibrate estimates of infection risk and better account for differential exposure and testing behaviors. Notably, our earlier work on COVID-19 demonstrated that, after adjusting for testing rates, the highest risk for infection was observed among younger males—contrary to patterns seen in unadjusted case data, which suggested higher risk among older females [9]. This supports the validity of our approach and highlights how differential testing and exposure behaviors can influence apparent infection risk. We used meta-regression rather than linear regression to allow for the incorporation of variance as weights in our models. This approach enabled more robust estimation by giving greater weight to observations with lower variance and appropriately accounting for uncertainty.

Nevertheless, there are limitations. Adjusted cases are not direct estimates of true infection incidence, but rather the number of infections that would have been observed if testing rates had been uniform across groups. Even in the most frequently tested group (reference), this does not imply that all infected individuals were tested. Asymptomatic or mildly symptomatic cases may go undetected, meaning the actual infection rates may still be underestimated.

In conclusion, this test-adjusted approach suggests a substantial proportion of pertussis cases go undiagnosed, particularly in preschool-aged children and the elderly. These groups may play a critical role in sustaining transmission and driving further outbreaks.

## Supplementary Information

Below is the link to the electronic supplementary material.

Supplementary Material 1

## Data Availability

The data that support the findings regarding population distribution of Ontario of this study are openly available in Statistics Canada Census, Population estimates on July 1, by age and gender, at: https://www150.statcan.gc.ca/t1/tbl1/en/tv.action?pid=1710000501%26pickMembers%255B0%255D=1.7%26pickMembers%255B1%255D=2.1%26cubeTimeFrame.startYear=2011%26cubeTimeFrame.endYear=2024%26referencePeriods=20110101%252C20240101. Pertussis cases and testing data that support the findings of this study are available from the corresponding author upon reasonable request.

## Abbreviations

CI: Confidence interval
DTaP: Diphtheria, tetanus, and acellular pertussis-containing vaccine
GTA: Greater Toronto area
IRR: Incidence rate ratio
PCR: Polymerase chain reaction
SIR: Standardized infection ratio
STR: Standardized testing ratio
