# Correction: Test-adjusted estimation for pertussis incidence in greater Toronto, Canada, 1993–2006

**DOI:** 10.1186/s12879-026-13692-x

**Published:** 2026-06-01

**Authors:** Clara Eunyoung Lee, Amy A. Howe, Natalie J. Wilson, Alicia A. Grima, David N. Fisman

**Affiliations:** Dalla Lana School of Public Health, University of Toronto, 155 College Street, Toronto, M5T 3M7 Canada

**Correction: BMC Infectious Diseases (2025) 26:79**

10.1186/s12879-025-12254-x

In the sentence beginning 'After adjustment, the 2-4-year group showed the highest relative incidence' in this article, the value (IRR: 5.503, 95% CI: 2.121–14.281) should have read '(IRR: 1.811, 95% CI: 1.117–2.938)'.

The original article has been corrected.

