# Supplementary material for: Test-adjusted estimation for pertussis incidence in greater Toronto, Canada, 1993–2006
Source: BMC Infect Dis. 2025 Dec 12;26:79. doi: 10.1186/s12879-025-12254-x (PMC12817766; doi:10.1186/s12879-025-12254-x)
Supplement: Supplementary file 1 — Supplementary Material 1 [file 12879_2025_12254_MOESM1_ESM.docx]

Suppl. Table 1. The estimated number of undiagnosed pertussis cases per reported case

| **Sex** | **Age group** | **Undiagnosed case per reported case** |
| --- | --- | --- |
| **Male** | < 1 y | 1 |
|  | 1 y | 2.111 (1.171-3.051) |
|  | 2-4 y | 13.690 (5.913-21.467) |
|  | 5-9 y | 6.835 (4.482-9.188) |
|  | 10-19 y | 3.180 (2.315-4.045) |
|  | 20-39 y | 0.939 (0.726-1.152) |
|  | 40-59 y | 0.612 (0.352-0.871) |
|  | 60-79 y | 1.051 (0.150-1.952) |
|  | ≥80 y | 4.257 (1.185-7.329) |
| **Female** | < 1 y | 1.179 (0.984-1.374) |
|  | 1 y | 3.463 (0.755-6.172) |
|  | 2-4 y | 6.476 (3.670-9.281) |
|  | 5-9 y | 5.002 (3.539-6.466) |
|  | 10-19 y | 6.802 (4.684-8.917) |
|  | 20-39 y | 3.616 (2.721-4.515) |
|  | 40-59 y | 2.812 (1.304-4.320) |
|  | 60-79 y | 1.222 (0.769-1.674) |
|  | ≥80 y | 5.184 (0.444-9.924) |

The parentheses indicate a 95% confidence interval.
